# Plasma expression levels of microRNA-101 are downregulated in patients with Parkinson’s disease

**DOI:** 10.1186/s13104-025-07604-6

**Published:** 2025-12-10

**Authors:** Tomohiro Omura, Hiroki Nishiguchi, Haruka Kaneda, Yumi Kitahiro, Kotaro Itohara, Kazuhiro Yamamoto, Toshiyasu Sakane, Ikuko Yano

**Affiliations:** 1 Department of Pharmacy, Kobe University Hospital, 7-5-2 Kusunoki-cho, Chuo-ku, Kobe, 650-0017 Japan; 2 Department of Integrated Clinical and Basic Pharmaceutical Sciences, Faculty of Medicine, Dentistry and Pharmaceutical Sciences, Okayama University, 2-5-1, Shikata-Cho, Kita-Ku, Okayama, 700-8558 Japan; 3 Department of Pharmaceutical Technology, Kobe Pharmaceutical University, Motoyamakita-Machi 4-19-1 Higashinada, Kobe, 658-8558 Japan

**Keywords:** Parkinson’s disease, microRNA, Endoplasmic reticulum stress, HRD1, SEL1L

## Abstract

**Objective:**

Parkinson’s disease (PD) is a neurodegenerative disorder characterized by the loss of dopaminergic neurons. Endoplasmic reticulum (ER) stress contributes to PD pathogenesis, with the ER-associated degradation (ERAD) system playing a protective role. HRD1, an ER-resident ubiquitin ligase, and its stabilizer SEL1L are involved in ERAD and neuronal survival. This study investigated alterations in the plasma levels of microRNA-101 (miR-101), which targets SEL1L, in patients with PD, and its potential role as a biomarker.

**Results:**

Plasma miR-101 levels in patients with PD significantly decreased compared with those in healthy controls. Receiver operating characteristic curve analysis demonstrated that miR-101 could moderately discriminate patients with PD from healthy individuals (area under the curve = 0.781, 95% confidence interval = 0.547–1.00). The optimal cutoff, as determined by the Youden index, was 0.737 (expression ratio relative to that in the healthy control group), yielding a sensitivity of 50% and specificity of 100%. These results suggested that reduced plasma miR-101 expression may reflect the pathophysiological state of PD, and miR-101 has potential as minimally invasive biomarker for PD.

## Introduction

Parkinson’s disease (PD) is a progressive neurodegenerative disorder and the second most common condition after Alzheimer’s disease [1, 2]. It is characterized by the degeneration of dopaminergic neurons in the substantia nigra, thereby leading to decreased dopamine levels in the striatum [3]. Dopaminergic drugs are typically used for symptomatic treatment. However, their long-term use is associated with significant adverse effects [4]. Increasing evidence suggests that endoplasmic reticulum (ER) stress plays an essential role in PD pathogenesis, particularly in the death of dopaminergic neurons in the midbrain [5, 6].

ER stress occurs when various stresses disrupt protein folding, synthesis, and glycosylation, resulting in the accumulation of unfolded proteins in the ER, which induces cell death. In response, eukaryotic cells initiate the unfolded protein response (UPR), which helps mitigate ER stress by activating the ER-associated degradation (ERAD) system [7]. ERAD facilitates the clearance of misfolded proteins via retro-translocation into the cytoplasm, ubiquitination by the E3 ubiquitin ligase, and subsequent degradation by the 26 S proteasome [8, 9].

Ubiquitin ligase 3-hydroxy-3-methylglutaryl-coenzyme A reductase degradation 1 (HRD1) is involved in ER stress, and the suppressor/enhancer lin-12-like (SEL1L) has been identified as an HRD1 stabilizer [10, 11]. During ER stress, mammalian cells suppress cell death by triggering HRD1-/SEL1L-mediated protein degradation [11, 12]. A previous study found that HRD1 was localized in dopaminergic neurons in the substantia nigra pars compacta of the midbrain [13]. In addition, another study showed that HRD1 degraded the parkin-associated endothelin receptor-like receptor (Pael-R), a substrate of the ubiquitin ligase parkin [14], thereby suppressing Pael-R-induced cell death [15]. Loss of parkin function in autosomal recessive juvenile PD results in the accumulation of unfolded Pael-R, which induces ER stress-mediated neuronal death [14]. Furthermore, we showed that HRD1 alleviated neuronal cell death in a cellular PD model using 6-hydroxydopamine (6-OHDA), which is widely used in in vitro and in vivo PD models [16, 17]. Conversely, cell death was enhanced even when the SEL1L expression was suppressed in a PD model [18], indicating an important role for SEL1L in PD pathogenesis.

MicroRNAs (miRNAs), a class of short noncoding RNAs, regulate gene expression post-transcriptionally, and they are implicated in various cellular processes and diseases, including PD [19–21]. Based on our previous study [22], miR-101 directly targets the SEL1L 3′ untranslated region, and a miR-101 mimic suppresses the 6-OHDA-induced increase in SEL1L expression and enhances cell death. Further, a miR-101 inhibitor suppressed this response.

Despite these findings, alterations in miR-101 expression in patients with PD remain unknown. Therefore, this study aimed to further elucidate the potential role of miR-101 in PD pathogenesis and its value as a diagnostic or therapeutic target by investigating its expression levels in the plasma of patients with PD.

## Methods and materials

### Human plasma samples

Caucasian plasma samples derived from eight patients with PD and six healthy donors were purchased from Analytical Biological Services Inc. (Wilmington, USA) via KAC Co., Ltd (Kyoto, Japan). Total RNA was extracted from 200-µL plasma using the miRNeasy Serum/Plasma Advanced Kit (QIAGEN GmbH, Hilden, Germany) based on the manufacturer’s instructions. For miRNA quantitation, cDNA was synthesized from RNA with specific miRNA reverse transcriptase primers (Thermo Fisher Scientific, Waltham, USA) using the TaqMan MicroRNA Reverse Transcription kit (Thermo Fisher Scientific). Quantitative real-time polymerase chain reaction (qPCR) was performed according to the instructions of the StepOnePlus Real-Time PCR System (Thermo Fisher Scientific) with the TaqMan MicroRNA Assay kit (Thermo Fisher Scientific) for miRNA quantitation. Probes for miRNAs specific for hsa-miR-101 (assay ID 002253) and has-miR-16-5p (assay ID 000391) genes were purchased from Thermo Fisher Scientific. The has-miR-16-5p was utilized as an internal control to normalize miRNA expression.

### Statistical analysis

Quantitative data were expressed as the mean ± SEM. The statistical significance of difference between healthy controls and patients with PD was determined using a two-tailed *t*-test with Excel (Microsoft^®^ Excel^®^ LTSC MSO (16.0.14332.21031) 64-bit, Microsoft, Redmond, WA, USA). Group comparisons for age and BMI were performed using the Mann-Whitney U test. Correlations between BMI and miR-101 expression levels were assessed using Pearson’s correlation coefficient, and the results are reported as the coefficient of determination (r^2^). Correlation analyses were performed separately for healthy donors and patients with PD, as well as for the combined dataset. Statistical analyses for group comparisons and correlations were conducted using R (version 4.5.1). To evaluate the diagnostic performance of miR-101 expression in distinguishing patients with PD from healthy controls, receiver operating characteristic (ROC) curve analysis was conducted using the pROC package (version 1.18.5) in R. The area under the ROC curve (AUC) and its 95% confidence interval (CI) were calculated using DeLong method. The statistical significance of the AUC compared with the null hypothesis (AUC = 0.5) was also assessed using DeLong’s test. The optimal cutoff of miR-101 expression was determined using the Youden index, from which the sensitivity and specificity were derived. The ROC curve was plotted with specificity on the x-axis and sensitivity on the y-axis. However, specificity is displayed in descending order (from 1 to 0) by default in the pROC package to facilitate interpretation.

## Results

The median age of the healthy donor group (*n* = 6) was 65 years (range: 61–66 years). This group comprised three males and the remaining were females. The median age of the PD group (*n* = 8) was 71 years (range: 68 − 82 years), and this group included four males and four females. BMI did not differ significantly between healthy donors (27.2 ± 0.8 kg/m^2^) and PD patients (24.4 ± 1.0 kg/m^2^; *p* = 0.065, Mann-Whitney U test). None of the healthy donors had a history of smoking or any prior medical conditions. All patients with PD presented with resting tremor as a characteristic motor symptom and exhibited postural instability among other clinical features. Detailed medical histories of study participants and prescribed medications are summarized in Table 1.

**Table 1:** Clinical information of healthy donors (a) and patients with PD (b)

(a) Healthy donors				
	Age	BMI	Smoking history	Medical history
#1	61	27.7	No	No
#2	64	25.1	No	No
#3	66	24.4	No	No
#4	66	29.4	No	No
#5	65	28.3	No	No
#6	64	28.5	No	No

(b) Donors with PD					
	Age	BMI	Symptoms and signs	Medical history	Treatment
#7	68	22.9	Tremor at rest, postural disorders	Cardiosclerosis, atherosclerosis	No
#8	80	21	Tremor at rest, postural disorders	Coronary artery disease, hypertension	Levodopa/cardiodopa, Tolcapone, Rotigotine
#9	71	23.6	Tremor at rest, muscle rigidity	Coronary artery disease, cardiosclerosis, chronic prostatitis	No
#10	68	28.1	Tremor at rest, postural disorders	Chronic esophagitis, chronic bronchitis	No
#11	70	26.8	Tremor at rest, postural disorders	Duodenal ulcer	No
#12	82	20.6	Tremor at rest, postural disorders	Osteoarthritis	Levodopa/benserazide
#13	72	25.1	Tremor at rest, postural disorders	Chronic esophagitis, chronic gastritis	No
#14	68	27.3	Tremor at rest, postural disorders	Coronary artery disease, depression	No

**Fig. 1:**
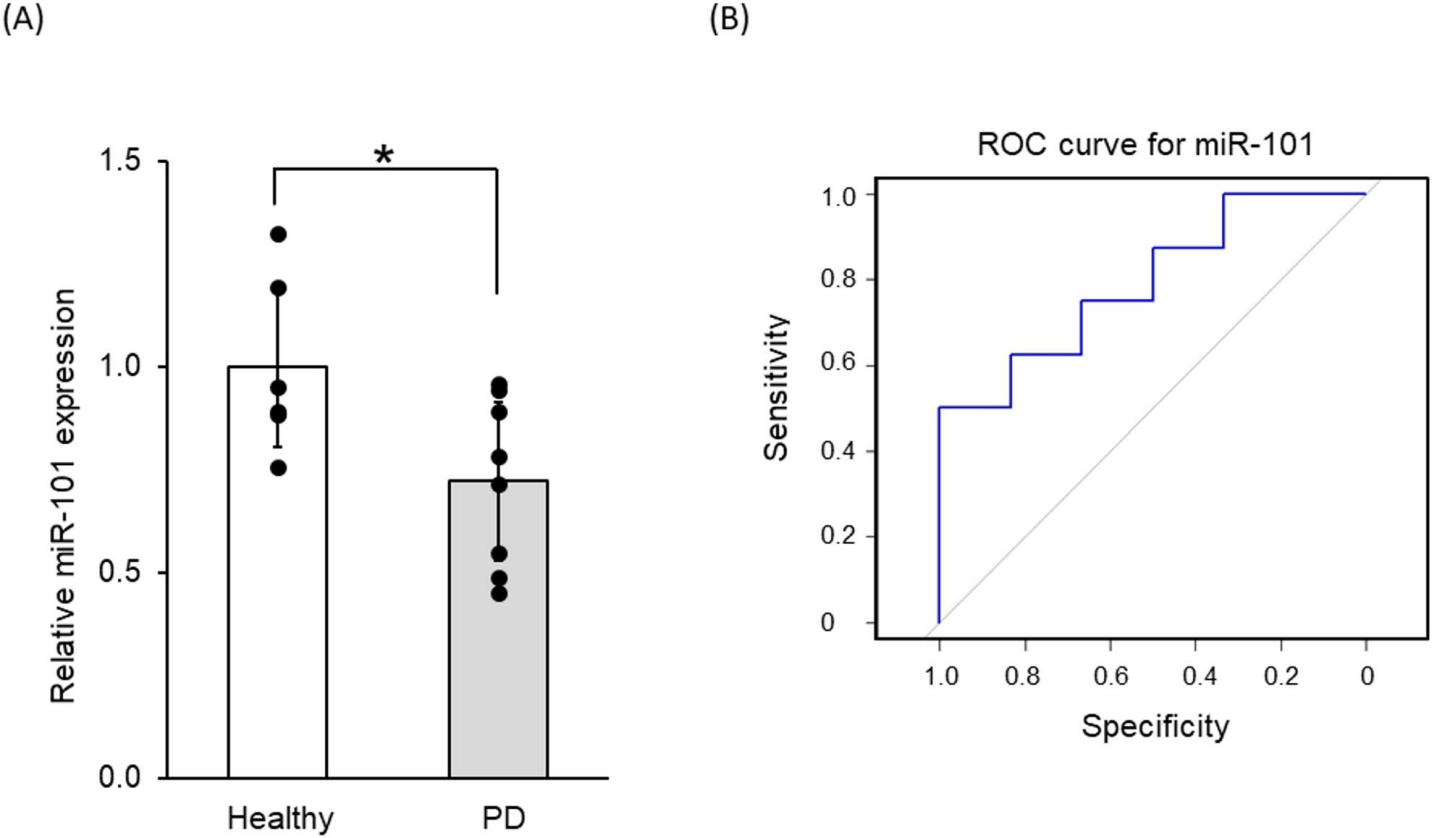
Real-time PCR analysis of miR-101 in samples from donors with PD and healthy donors. **A** Real-time PCR analysis of miR-101 in human plasma samples collected from healthy donors and patients with PD. Data on the miR-101 expression level relative to mean healthy value were expressed as the mean ± SEM. *p < 0.05 by the two-tailed *t*-test.** B** ROC curve for miR-101 expression in discriminating patients with PD from healthy controls. The x-axis represents specificity, which is plotted in reverse (from 1 to 0), and the y-axis represents sensitivity. Using the Youden Index, the optimal cutoff for miR-101 expression was identified as 0.737 relative to the mean healthy value, and the AUC was 0.781 (95% CI = 0.547–1.00)

We examined whether the expression levels of miR-101 changed in patients with PD.Results showed that the plasma expression levels of miR-101 in patients with PD significantly decreased compared with those in healthy donors (*p* = 0.030, Fig. 1A). ROC curve analysis was performed to evaluate the diagnostic performance of miR-101 expression in distinguishing patients with PD from healthy controls (Fig. 1B). The AUC was 0.781 (95% CI = 0.547–1.00). The difference from random classification (AUC = 0.5) was not statistically significant (*p* = 0.297, DeLong test). Using the Youden Index, the optimal cutoff of miR-101 expression was identified as 0.737 (expression ratio relative to healthy control group). At this threshold, the sensitivity was 50%, whereas the specificity was 100%.

**Fig. 2:**
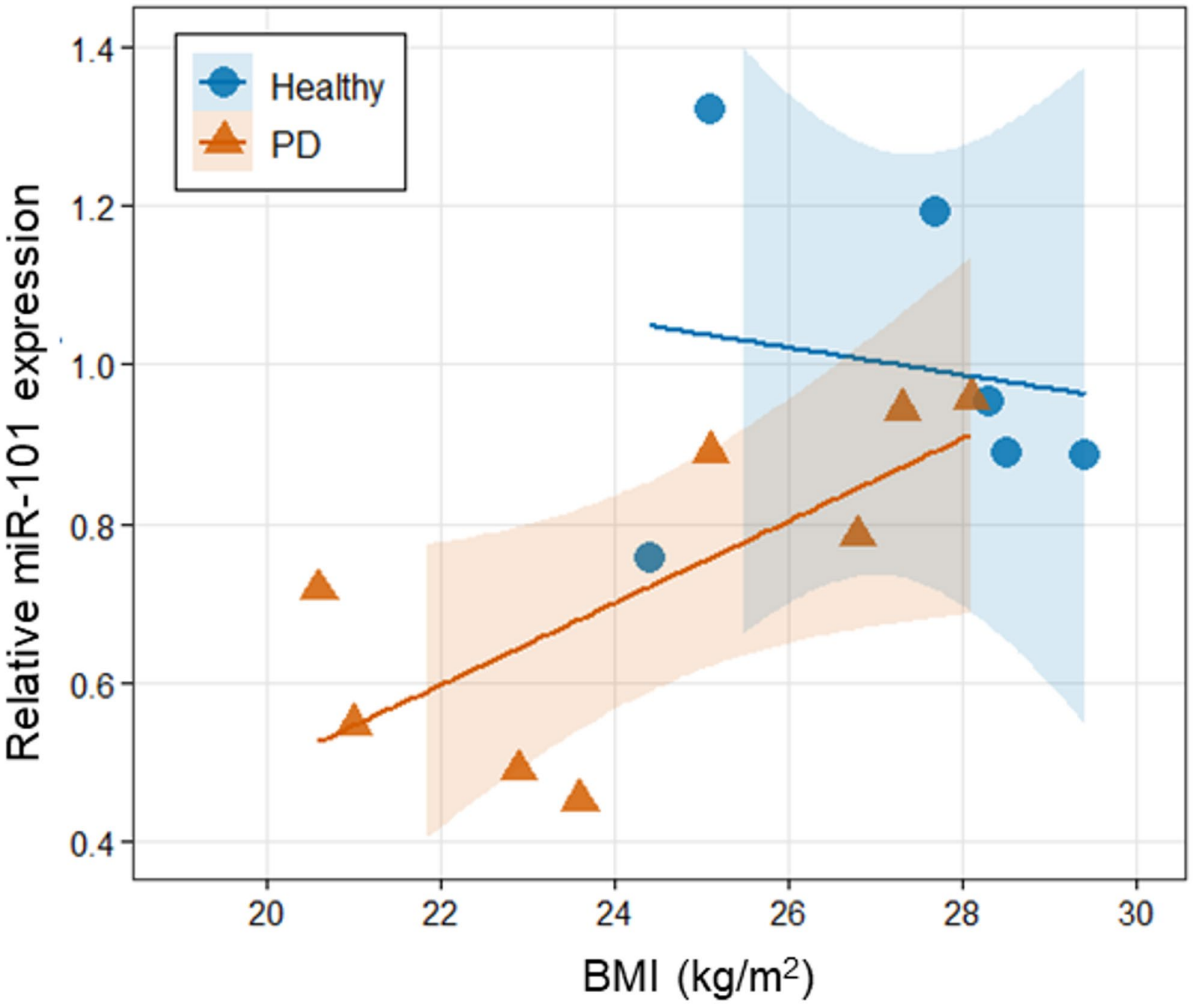
Correlation between BMI and miR-101 expression levels in healthy donors and patients with PD. Scatter plot presenting the relationship between BMI and relative miR-101 expression in healthy donors (*n* = 6, blue circles) and patients with PD (*n* = 8, orange triangles). Solid lines represent linear regression fits with 95% confidence intervals (shaded areas). Pearson’s correlation analysis: healthy donors, r^2^ = 0.026, *p* = 0.759; patients with PD, r^2^ = 0.520, *p* = 0.044; overall (*n* = 14), r^2^ = 0.340, *p* = 0.029

The results of Pearson’s correlation analysis between BMI and miR-101 expression are presented in Fig. 2. In healthy donors, no significant correlation between these variables was observed (r^2^ = 0.026, *p* = 0.759). In PD patients, a significant positive correlation was observed (r^2^ = 0.520, *p* = 0.044), indicating that BMI explains approximately 52% of the variance in miR-101 expression. When all samples were analyzed together, a significant correlation was observed (r^2^ = 0.340, *p* = 0.029).

## Discussion

Loss of dopaminergic neurons caused by ER stress is a possible pathogenic mechanism involved in the etiology of PD [6]. Mammalian cells activate the UPR to counteract ER stress, and the activities of the UPR system include the degradation and removal of unfolded proteins by activating the ubiquitin ligase in the ER [8, 9, 23]. A previous study investigated the association between HRD1 (ubiquitin ligase)/SEL1L (HRD1 stabilizer) and PD, and demonstrated that 6-OHDA-induced cell death is enhanced when the SEL1L expression is downregulated [18], which is in contrast to some findings showing that the HRD1 expression is downregulated.

This study showed that the plasma expression level of miR-101 was downregulated in patients with PD. ROC analysis suggested that miR-101 expression could represent a potential biomarker for PD. Although high specificity was observed in this study, the small number of healthy controls limits the generalizability of this finding. Therefore, the apparent ability to correctly identify individuals without PD should be interpreted with caution, and further validation in larger cohorts is warranted. Additionally, the moderate sensitivity limits the utility of miR-101 expression as a standalone diagnostic tool. The AUC indicated moderate discriminative ability; however, the wide 95% CI and lack of statistical significance likely reflect variability which can be attributed to the small sample size. Therefore, validation in larger cohorts is needed.

According to our previous study, miR-101 suppresses the expression of SEL1L, and miR-101 inhibition may have an inhibitory effect on neuronal cell death in a PD model [22]. Therefore, in patients with PD, the downregulation of miR-101 is believed to cause the upregulation of SEL1L in the brain, which may contribute to the suppression of dopaminergic neuronal cell death. However, the underlying mechanisms responsible for the reduced expression of miR-101 in PD remain to be elucidated.

Our analysis revealed that although PD patients had a numerically lower BMI than healthy donors, the difference was not statistically significant (24.4 ± 1.0 vs. 27.2 ± 0.8 kg/m²; *p* = 0.065), suggesting that the reduced miR-101 expression in PD patients is largely independent of BMI differences. Correlation analyses showed no significant correlation between BMI and miR-101 expression in healthy donors (r^2^ = 0.026, *p* = 0.759), whereas PD patients showed a significant positive correlation (r^2^ = 0.520, *p* = 0.044), indicating that BMI explains approximately 52% of the variance in miR-101 expression in PD patients. This finding suggests that BMI might modulate miR-101 expression specifically in PD patients. It is generally known that patients with PD tend to have lower BMI [24, 25], which has been suggested as a potential risk factor for PD. The positive correlation between BMI and miR-101 observed in this study warrants further investigation in larger cohorts.

Other research group have reported that the expression level of miR-101 in peripheral blood mononuclear cells (PBMCs) of patients with PD reduced compared with that in healthy individuals [26], consistent with the findings of the current study. Considering that the isolation of PBMCs and the extraction of miRNAs are technically demanding and time-consuming processes, the plasma-based miRNA extraction method used in this study may be a practical and less invasive approach for developing diagnostic tests for PD.

Several previous studies have shown that miR-16 is a reliable internal control for quantifying miRNA expression levels in the human plasma [27, 28]. Hence, miR-16 was used as the internal control in the current study. Although several other candidates were considered, our preliminary experiments using plasma samples showed that miR-16 had the highest stability (data not shown). Therefore, miR-16 is the most suitable internal control for this study, and it was employed accordingly.

In conclusion, plasma miR-101 may be a biomarker reflecting PD pathophysiology. Further, a decreased miR-101 expression in the brain has also been observed in Alzheimer’s disease [29], which is associated with ER stress [30]. Thus, miR-101 may have a broader potential as a biomarker for neurodegenerative diseases.

## Limitations

The current study had several limitations. First, the sample size was small. Second, the plasma samples were obtained from a commercial biorepository, and detailed clinical information including disease severity scores was not available because of the vendor’s data protection policies. Therefore, we could not assess the correlation between disease severity and miR-101 expression levels. Future studies with prospectively collected samples and comprehensive clinical assessments should investigate how miR-101 levels vary across different stages of PD severity, as assessed using scales such as the Hoehn and Yahr stage, to validate the clinical utility of miR-101 as a biomarker. Third, because this was a cross-sectional study, intra-individual longitudinal changes in miR-101 expression were not assessed. Therefore, it remains unclear whether the reduction in miR-101 expression reflects a pathophysiological change associated with disease progression or indicates pre-existing susceptibility to PD. Longitudinal studies are required to determine whether miR-101 acts as a state marker or a risk factor.

## Data Availability

The data analyzed during the current study are available from the corresponding author upon reasonable request.

## Abbreviations

6-OHDA: 6-hydroxydopamine
AUC: Area under the receiver operating characteristic
ER: Endoplasmic reticulum
ERAD: Endoplasmic reticulum-associated degradation
HMG-CoA: 3-hydroxy-3-methylglutaryl-coenzyme A
HRD1: 3-hydroxy-3-methylglutaryl-coenzyme A reductase degradation 1
Pael-R: Parkin-associated endothelin receptor-like receptor
PCR: Polymerase chain reaction
PD: Parkinson’s disease
ROC: Receiver operating characteristic
SEL1L: Suppressor/enhancer lin12 1-like
SEM: Standard error of the mean
UPR: Unfolded protein response
